# A Closed-Form Method of Acoustic Emission Source Location for Velocity-Free System Using Complete TDOA Measurements

**DOI:** 10.3390/s20123553

**Published:** 2020-06-23

**Authors:** Zilong Zhou, Yichao Rui, Xin Cai, Riyan Lan, Ruishan Cheng

**Affiliations:** School of Resources and Safety Engineering, Central South University, Changsha 410083, China; zlzhou@csu.edu.cn (Z.Z.); ruiyichao@csu.edu.cn (Y.R.); lanriyan.2018@gmail.com (R.L.); chengruishan@csu.edu.cn (R.C.)

**Keywords:** acoustic emission (AE), source location, time difference of arrival (TDOA), velocity-free

## Abstract

A closed-form method of acoustic emission (AE) source location for a velocity-free system using complete time difference of arrival (TDOA) measurements is proposed in this paper. First, this method established the governing equation of unknown acoustic velocity for each sensor; then, the governing equations of each of the three sensors were transformed into a linear equation, which can form a system of linear equations with the complete TDOA measurements. Third, the least squares solutions of the AE source coordinate and acoustic velocity were separately solved by an orthogonal projection operator. The proposed method was verified by the pencil-lead break experiment, and the results showed that the location accuracy and stability of the proposed method were better than those of traditional methods. Moreover, a simulation test was carried out to investigate the influence of noise scales on the location accuracy, and the results further prove that the proposed method holds higher noise immunity than the traditional methods.

## 1. Introduction

Acoustic emission (AE) location based on time difference of arrival (TDOA) between distinct sensor pairs has received increasing interest in recent years. It has been applied in numerous applications such as identifying hazard positions, monitoring structural integrity, and researching the mechanism of material damage [1,2,3,4,5,6,7,8,9,10]. With ceaseless research over several decades, numerous location methods based on TDOA measurements have been proposed to determine the position of an AE source [11]. Among them, the majority of these methods need to pre-measure the acoustic velocity of the media such as the Geiger-based method [12,13], simplex-based method [14,15], spherical interpolation method [16], and weighted least squares method [17,18]. These methods can achieve a good positioning result only when the acoustic velocity is accurate enough. However, in complex and variable engineering environments, the acoustic velocity might contain a degree of uncertainty or even unknown, which greatly limits the applications of traditional methods with pre-measured acoustic velocity [13,19].

To reduce the influence of the measurement error of the acoustic velocity, the AE source location methods for a velocity-free system have gained increasing attention [20]. The iterative location method is one of the most popular methods, which determine the AE source by minimizing the nonlinear cost functions. Aki et al. [21,22] first proposed the joint inversion of the source and the velocity structure by considering the horizontal inhomogeneity of the Earth’s interior. Ciampa et al. [23] presented the AE localization method for anisotropic plates and isotropic plates, which could determine both the AE source and group velocity using six sensors. Li et al. [24] constructed the cost function based on the time-difference-quotient principle, the minimum value of which was searched by the simplex method to obtain the spatial coordinate of the AE source. Dong et al. [25] proposed the source location method using the arrival times of both transverse and longitudinal waves for a three-dimensional micro-seismic source. All the above-mentioned methods can avoid the influence of measurement error of the acoustic velocity and improve the location accuracy. However, the minimization of the highly nonlinear cost function is sensitive to initial conditions, so there is no guarantee of the global convergence. 

Without the initial guess and the local convergence, the closed-form methods are more favorable. Dong et al. [26] gave an exact closed-form solution of a velocity-free system using six sensors. However, these methods cannot make full use of the extra sensors, when they are available, to improve the location accuracy. Based on this method, Dong et al. [27] further proposed the comprehensive analytical method, which could fully use all the available sensors. However, the assumption of the classic logistic density distribution on AE source coordinates is not optimal, resulting in a large location deviation. Without assuming the coordinate distribution, Zhou et al. [28] proposed a location method using a tri-variate kernel density estimator, which could achieve good location accuracy and stability in the presence of TDOA outliers. However, in most scenarios, the TDOA measurements do not have the outliers and only contain the random errors. In these scenarios, this method does not have advantages, and its positioning accuracy is not optimal. Moreover, due to the intensive computation of preliminary positionings, this method has poor real-time performance, especially in the case of many sensors. By exploring the linear least squares criterion, the United States Bureau of Mines method (USBM) can locate the AE source in real-time [29,30]. This method can also fully use the extra sensors and has higher location accuracy than the method using the exact number of sensors [26], but the location result is still highly biased because of the construction of an unfit cost function. To this end, a non-iterative location method of an unknown velocity system (NIUV) employing an improved spherical least squares criterion was further proposed [31,32,33]. The NIUV method not only holds good real-time performance, but also has higher positioning accuracy than the other methods. However, this method requires an a priori solution of squared velocity from a cubic equation, which may not exist or be unique. Moreover, the above-mentioned methods have the following two main limitations. One is that the use of the minimal TDOA set for locating an AE source is unreasonable and will lead to a large location error in a noise environment [34]. Another is that the coefficient matrix of these linear equations always has a large condition number and tends to be ill-conditioned because the elements in the coefficient matrix can differ by several orders of magnitude [35]. 

To solve the above problems, a closed-form method of AE source location for a velocity-free system using complete TDOA measurements is proposed. In this paper, the nonlinear governing equations were first transformed into a set of linear equations with complete TDOA measurements. Then, an orthogonal projection operator was introduced to reduce the ill-condition of this linear system. Finally, the pencil-lead break experiment and simulation test were conducted to verify its effectiveness and the location accuracy. 

## 2. Method 

### 2.1. Complete Time Difference of Arrival (TDOA) Measurements

The TDOA measurement is the most important input data for locating the AE source. It is defined as the difference in the arrival times measured at a pair of sensors. Let $\Delta t_{i,j}$ denote the TDOA; $t_{i}$ and $t_{j}$ denote the arrival times triggered by sensor i and j, so $\Delta t_{i,j}=t_{j}-t_{i}$. Given $\Delta t_{i,j}$ for *i* and *j* between 1 and *n*, the arrangement of the TDOA measurements is shown in a matrix as

(1) $$\left[\begin{array}{lllll} 0 & \Delta t_{2,1} & \cdots & \Delta t_{n-1,1} & \Delta t_{n,1}\\ \Delta t_{1,2} & 0 & \cdots & \Delta t_{n-1,2} & \Delta t_{n,2}\\ \vdots & \vdots & \ddots & \vdots & \vdots \\ \Delta t_{1,n-1} & \Delta t_{2,n-1} & \cdots & 0 & \Delta t_{n,n-1}\\ \Delta t_{1,n} & \Delta t_{2,n} & \cdots & \Delta t_{n-1,n} & 0 \end{array}\right],$$

The elements of $\Delta t_{i,i}$ on the main diagonal are equal to zero and the elements in the upper triangle are equivalent to that in the lower triangle ($\Delta t_{i,j}=-\Delta t_{j,i}$). Therefore, the n(n−1)/2 TDOA measurements in the lower triangle are sufficient to determine the TDOA matrix [36]. These TDOA measurements are defined as the complete TDOA set, as shown in the bold type in matrix (1). 

Compared with the complete TDOA set, the so-called minimal TDOA set defined in this paper is a subset of the complete TDOA set and only contains n−1 TDOAs measured from the sensor pairs with a common reference sensor as

(2) $$\left[\begin{array}{lllll} 0 & \Delta t_{2,1} & \cdots & \Delta t_{n-1,1} & \Delta t_{n,1}\\ \Delta t_{1,2} & 0 & \cdots & \Delta t_{n-1,2} & \Delta t_{n,2}\\ \vdots & \vdots & \ddots & \vdots & \vdots \\ \Delta t_{1,n-1} & \Delta t_{2,n-1} & \cdots & 0 & \Delta t_{n,n-1}\\ \Delta t_{1,n} & \Delta t_{2,n} & \cdots & \Delta t_{n-1,n} & 0 \end{array}\right],$$

The minimal TDOA set is printed in a bold type in the first column of the lower triangle. The minimal TDOA set can generate a “minimal spanning subtree”, which is enough to determine all the rest measurements in matrix (1). However, this statement holds only under an ideal environment (i.e., the error-free TDOA measurements and the exact known acoustic velocity) [34]. In actual engineering practice, the errors in the TDOA measurements and acoustic velocity are inevitable; the location result using the minimal TDOA set always has a large deviation. To increase the noise immunity, the complete TDOA measurements are used in this paper.

### 2.2. Construction of Linear Equations Using Complete TDOA Measurements 

Let $S_{i}\;\left(x_{i},\;y_{i},\;z_{i}\right)$ and (x,y,z) denote the coordinates of the AE sensor and AE source. The governing equation of velocity-free system for sensor *i* can be expressed as

(3) $$\sqrt{{\left(x_{i}-x\right)}^{2}+{\left(y_{i}-y\right)}^{2}+{\left(z_{i}-z\right)}^{2}}=v\left(t_{i}-t_{0}\right),i=1,\;2,\cdots ,n,$$

where $t_{0}\;$is the event time of the AE source, and v is the acoustic velocity that is treated as an unknown quantity rather than an input data that should be measured beforehand. 

The above governing equations are the nonlinear equations of arrival measurements, each of the three can be transformed into a linear equation of the TDOA measurements [37]. Randomly selecting three sensors i, j, and k from a multiple sensor system for analyses, which can form three TDOA measurements of $\Delta t_{j,k}$, $\Delta t_{k,i}$, and $\Delta t_{i,j}$. A single linear equation of AE source position can be constructed based on these three TDOA measurements, and the specific derivation processes deduced as follows: 

First, squaring and expanding the governing equations for sensors i, j, and k from Equation (3), we can get

(4a) $$v^{2}{\left(t_{i}-t_{0}\right)}^{2}={\left(x_{i}-x\right)}^{2}+{\left(y_{i}-y\right)}^{2}+{\left(z_{i}-z\right)}^{2},$$

(4b) $$v^{2}{\left(t_{j}-t_{0}\right)}^{2}={\left(x_{j}-x\right)}^{2}+{\left(y_{j}-y\right)}^{2}+{\left(z_{j}-z\right)}^{2},$$

(4c) $$v^{2}{\left(t_{k}-t_{0}\right)}^{2}={\left(x_{k}-x\right)}^{2}+{\left(y_{k}-y\right)}^{2}+{\left(z_{k}-z\right)}^{2},$$

where $L_{i}=x_{i}^{2}+y_{i}^{2}+z_{i}^{2}$. By subtracting Equation (4a) from Equations (4b) and (4c), the following expressions are obtained

(5a) $$v^{2}\Delta t_{j,i}\left(t_{j}+t_{i}\right)-2v^{2}\Delta t_{j,i}t_{0}=2\left(x_{i}-x_{j}\right)x+2\left(y_{i}-y_{j}\right)y+2\left(z_{i}-z_{j}\right)z+L_{j}-L_{i},$$

(5b) $$v^{2}\Delta t_{k,i}\left(t_{k}+t_{i}\right)-2v^{2}\Delta t_{k,i}t_{0}=2\left(x_{i}-x_{k}\right)x+2\left(y_{i}-y_{k}\right)y+2\left(z_{i}-z_{k}\right)z+L_{k}-L_{i}.$$

Next,

(6a) $$v^{2}\left(t_{j}+t_{i}-2t_{0}\right)=\frac{2\left(x_{i}-x_{j}\right)}{\Delta t_{j,i}}x+\frac{2\left(y_{i}-y_{j}\right)}{\Delta t_{j,i}}y+\frac{2\left(z_{i}-z_{j}\right)}{\Delta t_{j,i}}z+\frac{L_{j}-L_{i}}{\Delta t_{j,i}},$$

(6b) $$v^{2}\left(t_{k}+t_{i}-2t_{0}\right)=\frac{2\left(x_{i}-x_{k}\right)}{\Delta t_{k,i}}x+\frac{2\left(y_{i}-y_{k}\right)}{\Delta t_{k,i}}y+\frac{2\left(z_{i}-z_{k}\right)}{\Delta t_{k,i}}z+\frac{L_{k}-L_{i}}{\Delta t_{k,i}}.$$

Subtracting Equation (6b) from Equation (6a), this process generates

(7) $$\begin{array}{c} v^{2}\Delta t_{j,k}=2x\left(\frac{x_{i}-x_{j}}{\Delta t_{j,i}}-\frac{x_{i}-x_{k}}{\Delta t_{k,i}}\right)+2y\left(\frac{y_{i}-y_{j}}{\Delta t_{j,i}}-\frac{y_{i}-y_{k}}{\Delta t_{k,i}}\right)\\ +2z\left(\frac{z_{i}-z_{j}}{\Delta t_{j,i}}-\frac{z_{i}-z_{k}}{\Delta t_{k,i}}\right)+\frac{L_{j}-L_{i}}{\Delta t_{j,i}}-\frac{L_{k}-L_{i}}{\Delta t_{k,i}}. \end{array}$$

Multiplying both sides of Equation (7) by $t_{j,i}$ and $t_{k,i}$ to remove the denominator term,

(8) $$\begin{array}{c} -v^{2}\Delta t_{j,k}\Delta t_{k,i}\Delta t_{i,j}=2x\left[\Delta t_{k,i}\left(x_{i}-x_{j}\right)-\Delta t_{j,i}\left(x_{i}-x_{k}\right)\right]+2y\left[\Delta t_{k,i}\left(y_{i}-y_{j}\right)-\Delta t_{j,i}\left(y_{i}-y_{k}\right)\right]\\ +2z\left[\Delta t_{k,i}\left(z_{i}-z_{j}\right)-\Delta t_{j,i}\left(z_{i}-z_{k}\right)\right]+\Delta t_{k,i}\left(L_{j}-L_{i}\right)-\Delta t_{j,i}\left(L_{k}-L_{i}\right). \end{array}$$

Finally, the linear TDOA equation is obtained by simplifying and arranging Equation (8),

(9) $$e_{i,j,k}=a_{i,j,k}x+b_{i,j,k}y+c_{i,j,k}z+d_{i,j,k}v^{2},$$

where

$a_{i,j,k}=2\left(\Delta t_{j,k}x_{i}+\Delta t_{k,i}x_{j}+\Delta t_{i,j}x_{k}\right)$,
$b_{i,j,k}=2\left(\Delta t_{j,k}y_{i}+\Delta t_{k,i}y_{j}+\Delta t_{i,j}y_{k}\right)$,
$c_{i,j,k}=2\left(\Delta t_{j,k}z_{i}+\Delta t_{k,i}z_{j}+\Delta t_{i,j}z_{k}\right)$,
$d_{i,j,k}=-\Delta t_{j,k}\Delta t_{k,i}\Delta t_{i,j}$,
$e_{i,j,k}=\Delta t_{j,k}L_{i}+\Delta t_{k,i}L_{j}+\Delta t_{i,j}L_{k}$,

and Equation (9) becomes a linear equation by treating $v^{2}$ as a variable. 

For *n* sensors, a total of $C_{n}^{3}$ linear equations can be generated and written in matrix form as

(10) $$E=B\theta +Cv^{2},$$

where

$$E=\left[\begin{array}{c} e_{1,2,3}\\ \vdots \\ e_{i,j,k}\\ \vdots \\ e_{n-2,n-1,n} \end{array}\right],\;B=\left[\begin{array}{ccc} a_{1,2,3} & b_{1,2,3} & c_{1,2,3}\\ \vdots & \vdots & \vdots \\ a_{i,j,k} & b_{i,j,k} & c_{i,j,k}\\ \vdots & \vdots & \vdots \;\\ a_{n-2,n-1,n} & b_{n-2,n-1,n} & c_{n-2,n-1,n} \end{array}\right],\;C=\left[\begin{array}{c} d_{1,2,3}\\ \vdots \\ d_{i,j,k}\\ \vdots \\ d_{n-2,n-1,n} \end{array}\right],\;\mathrm{and}\;\theta =\left[\begin{array}{c} x\\ y\\ z \end{array}\right].\;$$

The complete TDOA measurements in matrix (1) have been fully utilized in these linear equations of Equation (10). 

### 2.3. Calculation of the Acoustic Emission (AE) Source Coordinate and Acoustic Velocity 

Since the TDOA errors cannot be known in advance, an equation residual ε is introduced into Equation (10):

(11) $$\varepsilon =E-B\theta -Cv^{2}.$$

However, the linear system Equation (11) is always ill-conditioned, because the elements in the coefficient matrixes *B* and *C* differ by several orders of magnitude [18]. Furthermore, unreasonable sensor placement can also cause the linear dependence of certain equations and lead to the ill-condition. The ill-condition of linear equations will result in the difficulty in solving equations and unstable positioning performance. To reduce the ill-condition of the linear system, an orthogonal projection operator $P_{B}^{⊥}$ is introduced to remove the components in the space spanned by the columns of *B*

(12) $$P_{B}^{⊥}=I-B{\left(B^{T}B\right)}^{-1}B^{T},$$

where $P_{B}^{⊥}$ is an idempotent projection matrix with the mathematical relationship of $P_{B}^{⊥}{}^{2}=P_{B}^{⊥}$; the symbols *T* and −1 at the upper-right corner represent the transpose and inversion of a matrix.

Multiplying Equation (11) by $P_{B}^{⊥}$ to remove the θ term, a new linear equation only with respect to $v^{2}$ can be obtained

(13) $$\varepsilon^{\prime }=P_{B}^{⊥}\varepsilon =P_{B}^{⊥}\left(E-Cv^{2}\right).$$

Solving Equation (13) for v,

(14) $$v={\left(\underset{\theta }{\min}\overset{n}{\underset{i=1}{\sum }}{\varepsilon_{i}^{\prime }}^{T}\varepsilon_{i}^{\prime }\right)}^{1/2}={\left[{\left(C^{T}P_{B}^{⊥}C\right)}^{-1}C^{T}P_{B}^{⊥}E\right]}^{1/2}.$$

When the minimizing v value is found, the least squares solution of θ given v can also be solved from Equation (11) by

(15) $$\theta =\underset{\theta }{\min}\overset{n}{\underset{i=1}{\sum }}\varepsilon_{i}^{T}\varepsilon_{i}={\left(B^{T}B\right)}^{-1}B^{T}\left(E-Cv^{2}\right).$$

Through the above calculations, the AE source coordinate can be readily determined. For the three-dimensional positionings, the proposed method requires more than or equal to six available sensors, while in two-dimensional panel positionings, the minimum number of sensors is reduced by one, and at least five sensors are required. 

**Figure 1:**
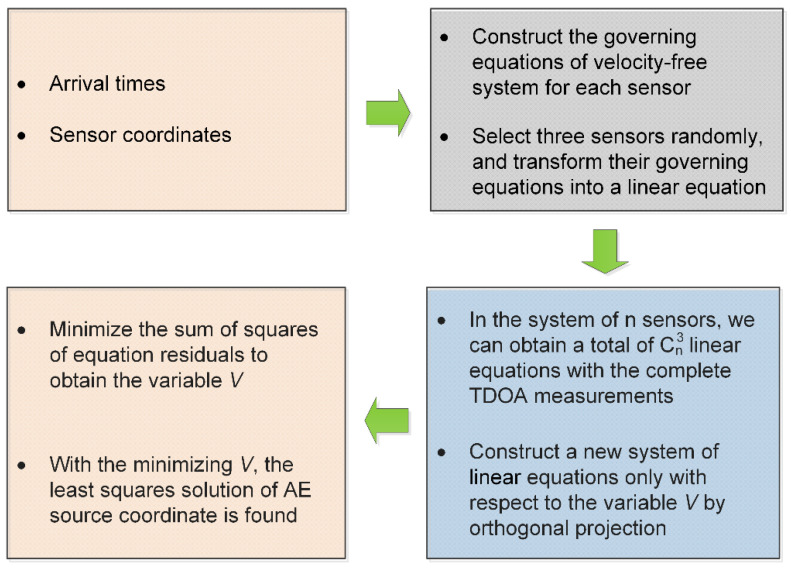
The entire location process of the proposed method.

The entire location process of the proposed method is shown in Figure 1. 

## 3. Experimental Verification 

**Figure 2:**
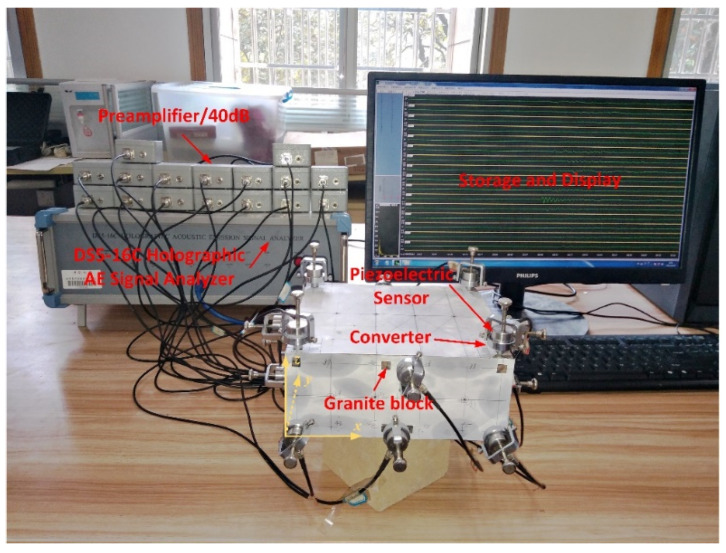
Photographic view of the acquisition process of the acoustic emission (AE) signal and experimental equipment (the white paper with the grid in this picture is to provide the coordinates of the lead-break points).

To verify the feasibility of the proposed method, the pencil-lead break experiment was performed on a granite block. The detailed acquisition process of AE signals and the experimental equipment are shown in Figure 2. This acquisition process can be divided into five steps. (1) The AE source was generated by the pencil-lead break, where the hard-black pencil lead was 0.5 mm in diameter, 4 mm in length, and broken at 30° of the contact surface; (2) the AE signal emitted from the source was received by the piezoelectric sensor and the frequency of the received signals ranged from 50 to 400 Hz; (3) the AE signal was amplified with the gain of 40 dB; (4) the amplified signal was collected by a DS5-16C Holographic AE Signal Analyzer, where the sampling frequency was set to 3 MHz to cover the signal frequency domain without distortion; and (5) the holographic AE signal was stored and displayed in the computer, and some post-processing could be performed. To determine the specific coordinates of the AE sources and sensors, the Cartesian coordinate system was established with a vertex of the block as the origin point, as shown in Figure 2. Then, the specific coordinates of the sources and sensors can be easily determined with the help of the coordinate grid lines on the white paper attached to the block. 

**Figure 3:**
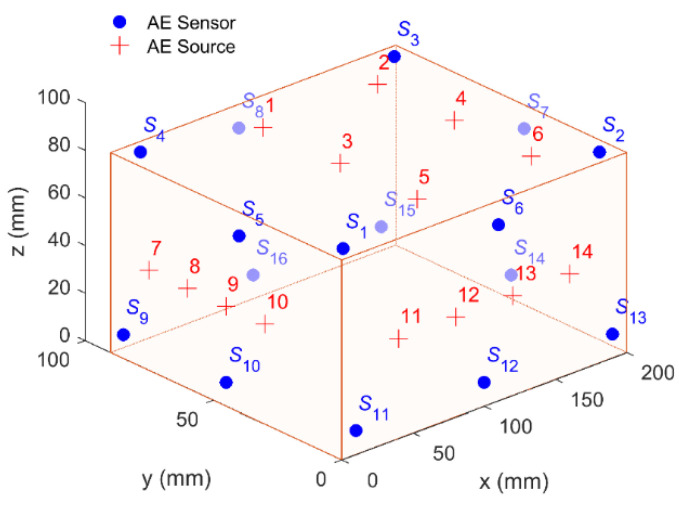
The layout of the AE sensors and sources in the pencil-lead break experimental equipment.

The placement of sensors has a great influence on the positioning results [38]. To achieve the optimal positioning effect, the sensors should surround the monitored area as scattered as possible. Figure 3 shows the layout of the AE sensors and sources. It can be seen that sixteen sensors were dispersedly mounted on the surface of the 200 mm × 179 mm × 84 mm block. Their coordinates were (10, 10, 84), (190,10, 84), (190, 170, 84), (12, 170, 84), (0, 80, 74), (110, 0, 74), (200, 80, 74), (90, 180, 74), (0, 170, 10), (0, 90, 10), (10, 0, 10), (100, 0, 10), (190, 0, 10), (200, 90, 10), (190, 180, 10), and (100, 180, 10) (in mm). In a real positioning system, the AE source may occur anywhere in the monitored area. The AE sources in different positions and directions may have different positioning accuracy. In order to better verify the positioning performance, we arranged fourteen AE sources generated by the pencil-lead breaks on three sides of the block. Their coordinates were (80, 150, 84), (160, 150, 84), (80, 90, 84), (160, 90, 84), (80, 30, 84), (160, 30, 84), (0, 150, 42), (0, 120, 42), (0, 90, 42), (0, 60, 42), (40, 0, 42), (80, 0, 42), (120, 0, 42), and (160, 0, 42) (in mm). 

**Figure 4:**
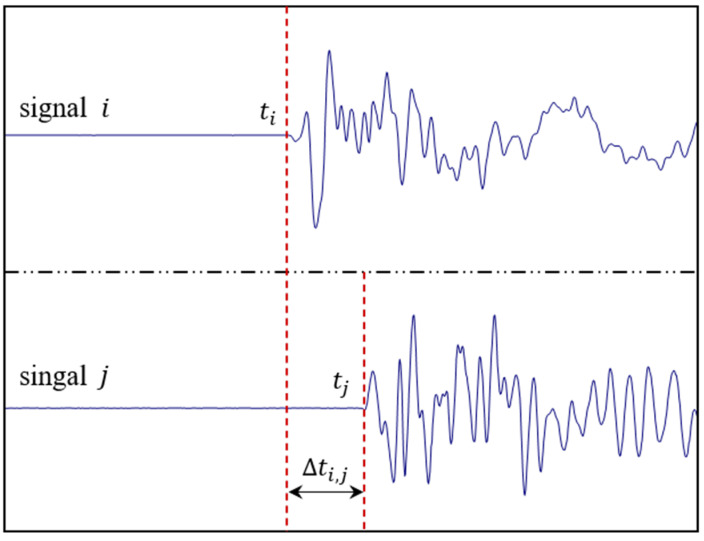
The schematic diagram of arrival time picking and the formation of TDOA measurements.

Before locating the AE source, the arrival time of each triggered sensor should be picked up first. The basic principle of arrival picking is to determine the corresponding time of the first obvious take-off point at the waveform, as shown in Figure 4. After obtaining the arrival time of each sensor, the TDOA measurements can be formed by subtracting the arrival time of sensor *j* from that of sensor *i*, (i.e., $\Delta t_{i,j}=t_{j}-t_{i}$). However, when a sensor fails, there will be an outlier in the TDOA measurements. The invalid sensors should be excluded in advance. There are two main characteristics of the invalid sensors: (1) the signal recorded by the invalid sensor is white noise or has low signal-noise-ratio; and (2) the recorded signal is irrelevant to the AE event to be located. After filtering the invalid sensors, the proposed method can locate the sources quite efficiently according to the TDOA measurements and the coordinates of the valid sensors. 

**Figure 5:**
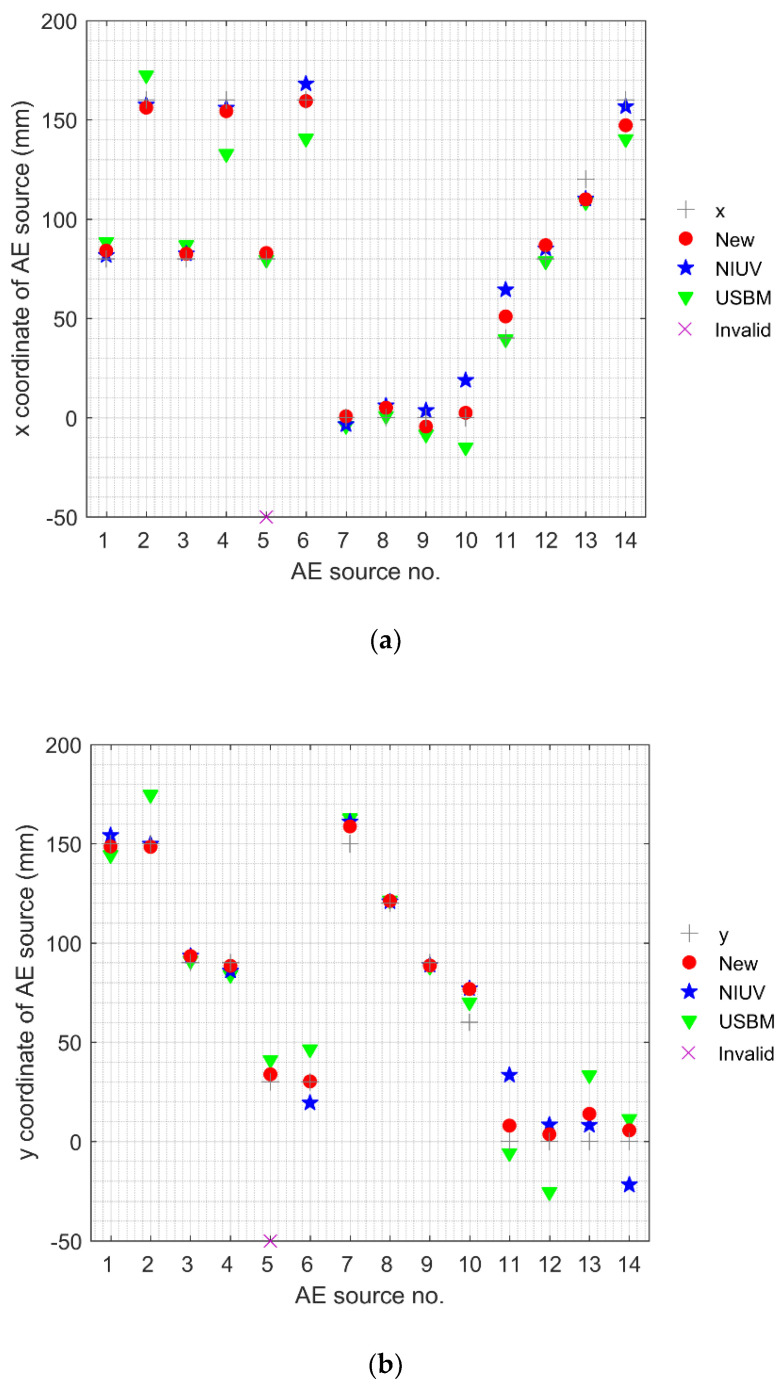

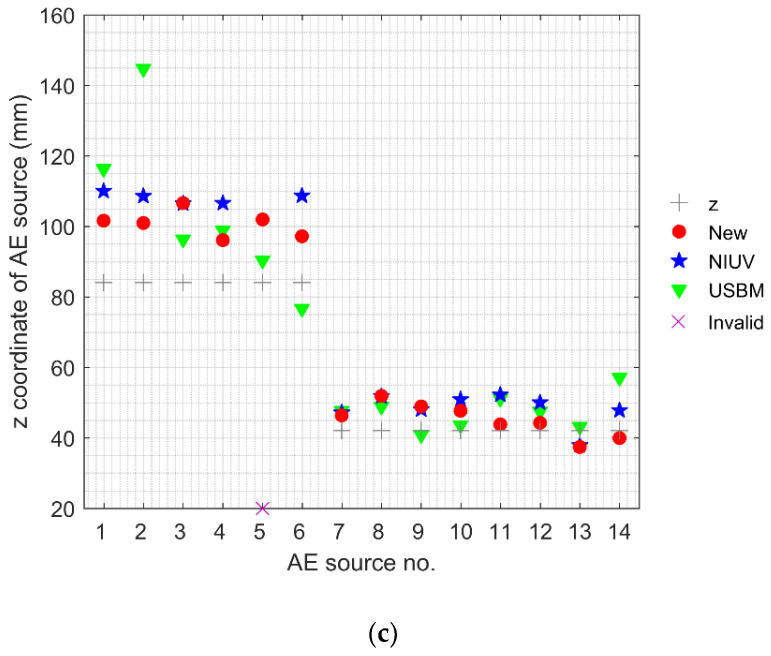
Upper graphs show the location results for AE sources no. 1 to no. 14 as determined by different methods, where graphs (**a**,**b**), and (**c**) show the x, y, and z coordinates, respectively.

**Figure 6:**
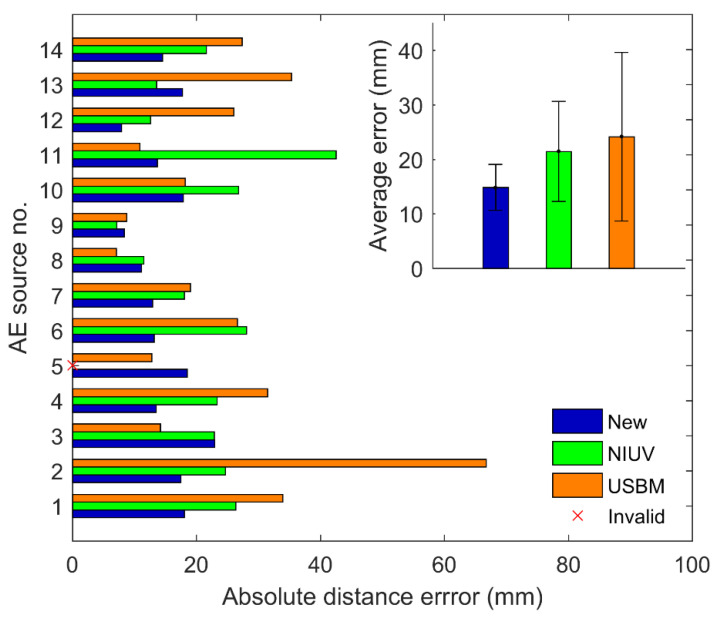
The absolute distance errors for AE sources no. 1 to No. 14, their average errors and standard deviations determined by different methods.

Figure 5 shows the *x*, *y*, and *z* coordinates of the AE sources determined by different methods. There was an invalid location of AE source no. 5 for the NIUV method. If one location has no solutions or multiple solutions, as described in the Section 1, then this location is defined as an invalid location (“Invalid” for short in all figures). From Figure 5, we can observe that the coordinates of the new method denoted by markers “●” are generally closer to the true AE source coordinates denoted by markers “+”, compared with the NIUV and USBM methods denoted by the markers “★” and “▼”. In other words, the location performance of the new method was better than that of the traditional methods. Figure 6 illustrates the absolute distance errors of 14 location results, their average errors, and the standard deviations for these three methods. From this figure, it can be seen that the absolute distance errors of the new method were higher than that of the NIUV method (expect AE source no. 3, 9, and 13) and the USBM method (expect AE source no. 3, 5, 8, and 11). Moreover, the lower average errors and the standard deviations of the new method further demonstrate that the new method has a higher location accuracy and stability than the traditional methods. 

## 4. Simulation Analysis 

**Figure 7:**
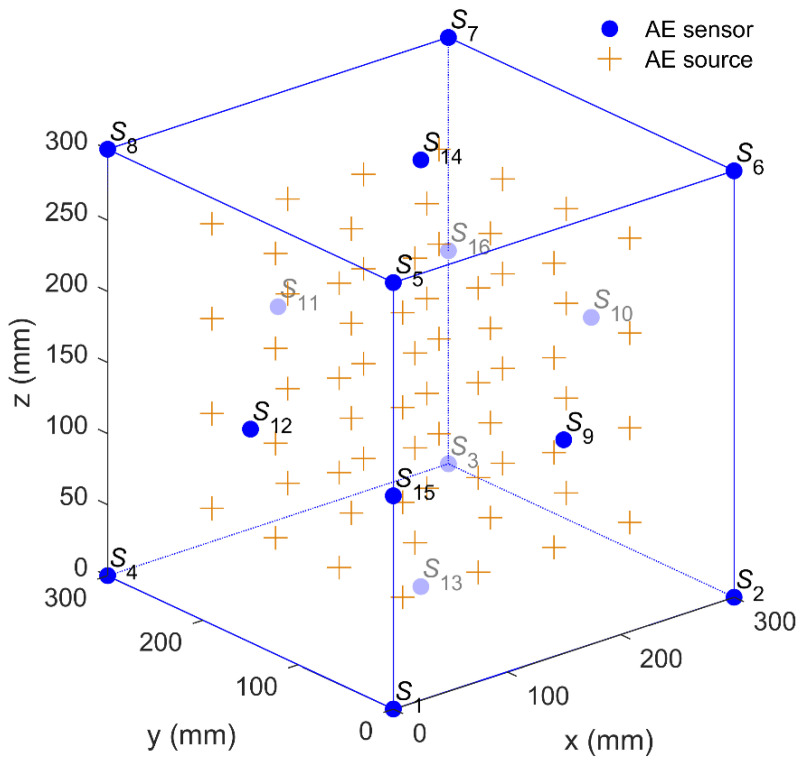
Layout of the AE sensors and the AE sources.

Due to the influence of environmental noises, the arrival times are often corrupted by the noises of different scales, which affects the location accuracy. The above pencil-lead break experiment verifies the feasibility of the new method, however, the noise scales in an opaque medium are uncontrollable. To further verify the influence of noise scales on location accuracy, a simulation test with controllable noise scales was applied to compare the location performance of different methods. Herein, a 300 mm × 300 mm × 300 mm cubic monitoring system was surrounded by 16 scattered sensors, and their coordinates were (0, 0, 0), (300, 0, 0), (300, 300, 0), (0, 300, 0), (0, 0, 300), (300, 0, 300), (300, 300, 300), (0, 300, 300), (150, 0, 150), (300, 150, 150), (150, 300, 150), (0, 150, 150), (150, 150, 0), (150, 150, 300), (0, 0, 150), and (300, 300, 150) (in mm). To verify the location performance of the AE source inside the monitoring area, a total of 64 (4×4×4) virtual AE sources were uniformly generated within the monitoring area, as shown in Figure 7. In order to study the influence of noise scales on the location performance of the proposed method, different errors with the standard deviations of 0.3, 0.6, 0.9, 1.2, and 1.5 μs were added to the arrival times as the noise disturbances.

**Figure 8:**
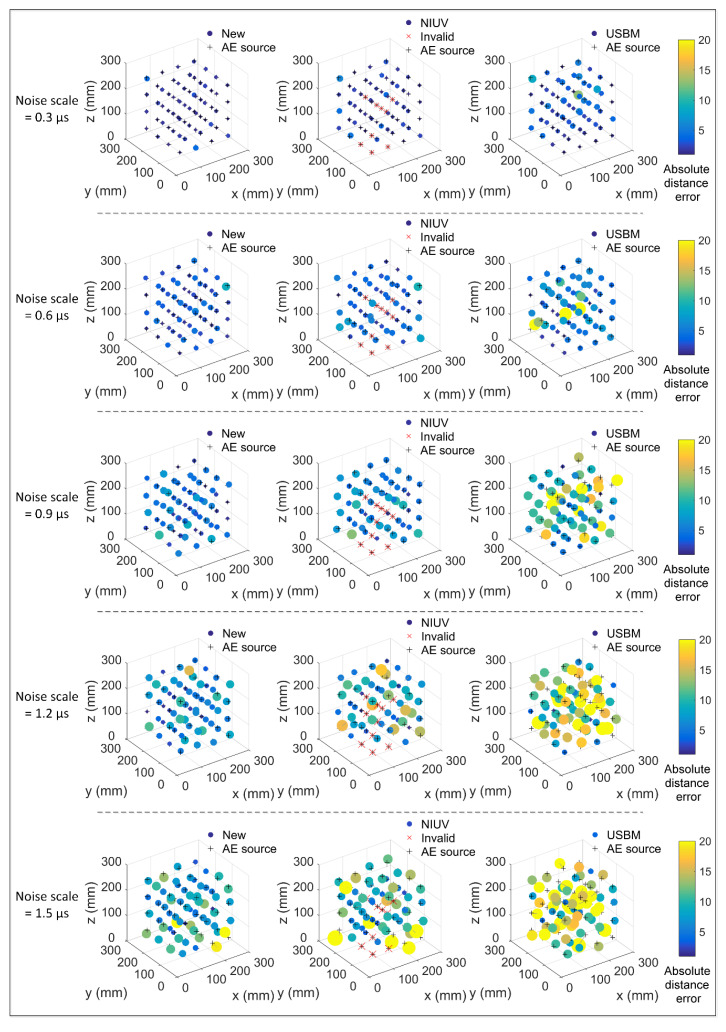
The spatial locating results for different sources as determined by the three methods under the noise scales of 0.3 μs, 0.9 μs, and 1.5 μs.

Figure 8 shows the three-dimensional location results for different sources determined by three methods under the noise scales of 0.3, 0.6, 0.9, 1.2, and 1.5 μs. The absolute distance error is characterized by the size and color of the sphere. The larger and brighter the sphere, the greater the location error. From Figure 8, we observed that under different noise scales, there were 9, 10, 10, 10, and 9 invalid locations for the NIUV method, while the new method and the USBM method had no such problems. Furthermore, the spheres for the NIUV and USBM methods are generally larger and brighter than those of the new method under different noise scales, which indicates that the new method has a higher positioning accuracy compared to the traditional methods. Although the location errors of the three methods rise with the increase of the noise scale, the positioning advantage of the new method is also more obvious. 

**Figure 9:**
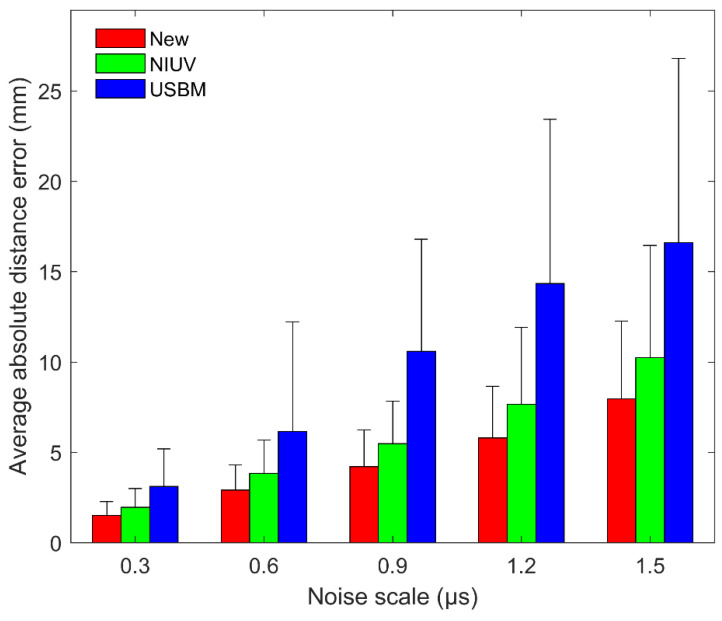
The average absolute distance errors and the standard deviations of 64 AE sources determined using three methods under different noise scales.

Figure 9 illustrates the average absolute distance errors and the standard deviations of the three methods under different noise scales. In this figure, the average absolute distance errors and the standard deviations of the NIUV method were calculated after filtering the invalid locations. It can be seen that the average absolute distance errors and the standard deviations of the new method were always smaller and increased more slowly than those of the NIUV and USBM methods, which shows that the proposed method had higher location accuracy and stability than the traditional methods. Therefore, the proposed method has higher noise immunity and is more suitable for noisy engineering environments.

## 5. Conclusions 

In this paper, a closed-form method of AE source location for a velocity-free system using complete TDOA measurements was proposed. The advantages of the proposed method are as follows: (1) the input data of acoustic velocity is not needed and an accurate acoustic velocity can be inversed in real-time when performing the source location; (2) this method utilizes the complete TDOA measurements with higher noise immunity, further improving the location accuracy and stability; (3) the event time is eliminated, reducing the degree of freedom and improving location accuracy; (4) without square root operation, the problem of no solutions and multiple solutions is avoided; and (5) by introducing the orthogonal projection operator, the ill-condition of the linear system is reduced. The pencil-lead break experiment was conducted to verify the location performance of the proposed method; the results showed that the proposed method had better positioning accuracy and stability than the traditional methods. The results of the simulation test under different noise scales demonstrated that the proposed method always had higher noise immunity than the traditional methods. However, the proposed method still has the following limitation: when there are outliers in the TDOA measurements, the location result of the proposed method will have a great deviation. Therefore, a positioning method that can automatically identify and filter the outliers is of great importance and worthy of further study.
